# Suicide attempt and its determinants among youth in central, north, and west Gondar zones, northwest Ethiopia: Findings from the youth health project

**DOI:** 10.3389/fpsyt.2022.1031402

**Published:** 2023-01-04

**Authors:** Alehegn Bishaw Geremew, Abebaw Addis Gelagay, Hedija Yenus Yeshitila, Telake Azale, Yohannes Aynaw Habitu, Solomon Mekonnen Abebe, Eshetie Melese Birru, Elsa Awoke Fentie

**Affiliations:** ^1^Department of Reproductive Health, College of Medicine and Health Science, Institute of Public Health, University of Gondar, Gondar, Ethiopia; ^2^Department of Health Education and Behavioural Sciences, College of Medicine and Health Sciences, Institute of Public Health, University of Gondar, Gondar, Ethiopia; ^3^Department of Human Nutrition, College of Medicine and Health Science, Institute of Public Health, University of Gondar, Gondar, Ethiopia; ^4^Department of Pharmacology, School of Pharmacy, College of Medicine and Health Science, University of Gondar, Gondar, Ethiopia

**Keywords:** suicide, attempt, determinants, youth, Ethiopia

## Abstract

**Introduction:**

Suicide is a deliberate attempt to take one's own life. Suicidal behavior among adolescents and young people, a significant global public health issue, is under-researched, particularly in developing nations like Ethiopia. Therefore, this study aimed to assess the prevalence of suicide attempts and their determinants among school-aged and out-of-school youth in the central, north, and west Gondar zones of Ethiopia.

**Methods:**

A community-based cross-sectional study was conducted from 15 March to 15 April 2019, among youth in the central, north, and west Gondar zones. The data for this study were extracted from information collected for the assessment of common health problems and risky behaviors among youth. A multistage cluster sampling technique was used to collect the data using face-to-face interviewer-administered questionnaires. Bivariable and multivariable logistic regression analyses were done to identify the independent determinants of suicide attempts. An adjusted odds ratio (AOR) with a 95% confidence interval (CI) was used to identify the presence and direction of the association between the dependent and independent variables.

**Results:**

A total of 1,597 youth participated in this study, and the mean age of the participants was 19.1 ± 2.8 standard deviations (SDs). The lifetime prevalence of suicide attempts in the study participants was 5.5% (95% CI: 4.4, 6.8%). Risky sexual practices (AOR = 1.89, 95%CI: 1.19–2.99), the presence of common mental health problems (AOR = 6.31, 95% CI: 3.78–10.88), having a personal income (AOR = 1.68, 95% CI: 1.05–2.71), and a history of violence (AOR = 2.81, 95% CI 1.76–4.49) were significantly associated with a suicide attempt.

**Conclusion:**

In this study, the prevalence of lifetime suicide attempts among youth is high. The presence of common mental health problems, having a personal income, risky sexual practices, and a history of violence increase the likelihood of suicide attempts. Working on the reduction of risky sexual practices, ensuring the integration of psychological treatment into medical treatment programs for victims of violence, psychosocial support for young people, and integrating youth-friendly health services to promote mental health would help to reduce suicide attempts among youth.

## Introduction

Suicide is the deliberate act of causing one's own death, and a suicide attempt is an intentional but unsuccessful attempt to take one's own life. Any self-reported passive or active desire to end one's life is suicidal ideation (1, 2). Young people commonly end their lives using poison, suffocation, and firearms. Most adolescents used hanging as a method of attempting suicide, with a higher tendency in men, but there was a higher tendency of poisoning in women (3, 4).

In 2019, suicide rates in Africa (11.2/100,000), Europe (10.5/100,000), and South-East Asia (10.2/100,000) were higher than the global average (9.0/100,000), and low- and middle-income nations (77%) accounted for the majority of suicide deaths (5).

A suicide attempt is the second leading cause of death for those between the ages of 15 and 29 years and accounts for 1.4% of the worldwide burden of pre-mature death (5). The incidence of suicidal ideation and attempts among adolescents in Dangla, Ethiopia was reported to be 22.5 and 16.2%, respectively (6), while the lifetime pooled prevalence of suicide attempts among Ethiopia's general population was determined to be 4% (3–6%) (7). Suicide and suicide attempts have major emotional, physical, and economic effects in addition to the loss of life. Suicide attempt survivors may experience serious injuries that might have a long-term negative effect on their health, including depression and other mental health issues. Additionally, when someone takes their own life, their surviving relatives and friends may go through shock, anger, guilt, depressive, or anxious symptoms, and may even have suicidal thoughts themselves (5, 6).

Different factors can play a role in the development of suicidal behavior. These include gender, loneliness, sadness, anxiety, depression, poor social support, being physically hurt, family history of suicide attempts, lifetime alcohol use, substance use, rural residence, less frequent engagement in religious practice, low monthly income, feeling neglected by parents, and having lost something valuable (6–9).

To prevent the tragedy of suicide from continuing to cost lives and affect many millions through the loss of loved ones or suicide attempts, the World Health Organization (WHO) has prioritized the reduction of suicide mortality as a global target and included it in both the WHO Mental Health Action Plan 2013–2030 and the 13th General Program of Work 2019–2023; however, suicide attempts continue to be a global public health issue (10).

Even though there have been studies conducted in Ethiopia regarding suicide attempts in young people, they are mainly institutionally based and either at the university level or high or preparatory school (6, 10–12), or only among patients with the human immunodeficiency virus (HIV) or people with severe mental disorders (13–16). As a result, in almost all public health research, youth suicide attempts are overlooked and exclude out-of-school youth, and adolescent populations in Ethiopia. Therefore, this community-based study aimed to assess the magnitude of suicide attempts and identify factors associated with suicide attempts in school-aged and out-of-school youth (15–24 years) in the central, north, and west Gondar zones, northwest Ethiopia. The result of this study may assist in preventing further social and economic consequences associated with suicide and suicide attempts. The data from this study provide important information that can be used to design appropriate interventions.

## Methods

### Study designs and period

A community-based cross-sectional study design was conducted among youth from 15 March to 15 April 2019.

### Study setting

This study was conducted in the central, west, and north Gondar zones, the Amhara regional state, northwest Ethiopia. According to each zonal administrative office report, a total of 24 woredas and one city administration are present in the three zones: there are 13 woredas in the central Gondar zone, seven in the north Gondar zone, and four in the west Gondar zone. The total number of kebeles in the three zones was 546. Based on the 2014–2017 GC population projection report, a total of 3,441,885 people reside within the three zones.

### Population

The source of the population was young people who reside in both rural and urban areas in the central, west, and north Gondar zones. Young people in the selected districts were the study population for this study. Those suffering from severe illness and unable to communicate for data collection were excluded.

### Variables of the study

The dependent variable in this study was lifetime suicide attempts. In addition, the independent variables were sociodemographic factors (age, sex, residency, religion, marital status, occupation, education status, and income), clinical and social factors (common mental health problems, chronic illness, feeling sad or worried, and risky sexual practices), and substance use characteristics (having previously drunk alcohol, chewed khat, or smoked a cigarette, or other substance use).

### Operational definitions

#### A lifetime suicide attempt

This is determined by the response to the question, “Have you ever tried to take your own life?” If the response is “yes,” the respondent has attempted suicide (17).

#### Suicidal ideation

If a respondent answers yes to the question “Have you ever seriously thought taking your own life?,” they are considered to have suicidal ideation (18).

#### Common mental health problems

We identified common mental health illnesses using WHO self-reported questionnaires 20 (SRQ-20) (19). Participants were considered to have a common mental health issue if they answered “yes” to more than seven of the 20 SRQ questions (20).

#### Risky sexual practices

This is when someone engages in sexual activity before the age of 18, has multiple sexual partners, engages in sexual activity under the influence of alcohol, engages in casual sex, or engages in sexual activity without using or with inconsistent use of a condom (21).

#### Violence

In this study, young people were defined as having experienced violence if at least one form of violence (emotional, sexual, or physical) had occurred in the previous 12 months.

#### Previous substance use

Defined as consuming alcohol, khat, or cigarettes, or other substances at least one time in his or her lifetime (22).

### Sample size and sampling techniques

The required sample size was determined using the single population proportion formula *n* = *za*^2^/2*p*(1–*p*)/*d*^2^ by considering an estimated 16.2% prevalence of suicide attempts among adolescents from a similar study conducted in Danigla town. In addition, by taking margin of error (*d*) as a 3 and 95% confidence interval (CI), design effect 2 and add a 10% non-response rate. Thus, the calculated final sample was 1,274. However, the predicted sample size was smaller than the data collected for the assessment of common health problems and contributing factors among youth in the central, north, and west Gondar zones in northwest Ethiopia. Therefore, all the data collected from eligible participants for this study were included in the analysis.

Study participants were recruited using a multistage cluster sampling technique. In the first stage five districts—two from the central Gondar zone (West Belessa and Alfa), two from the north Gondar zone (Janamora and Debark Zuria), and one from the west Gondar zone—Abrajira—were chosen through a lottery. Second, three kebeles (one urban and two rural) were randomly selected from each district and sub-city. Three clusters (ketena) were also randomly selected from each kebele. The total sample size was allocated equally for each district, and finally, eligible participants were interviewed when they were at the household or near the household during the data collection period. Only one individual was invited to participate by lottery when more than one eligible participant was found in a single household.

### Data collection tool and procedures

A structured interviewer-administered questionnaire was used to collect sociodemographic characteristics, reproductive health-related characteristics, common health morbidities, and other relevant factors. A total of 20 self-reported questions were used in the interview to measure the common mental health problems [24]. Participants who responded “yes” to each question were given a score of 1, and those who responded “no” were given a score of 0. Approximately 24 data collectors—women who were diploma nurses and midwives—were hired, and six supervisors with bachelor's degrees, one from each district, participated in the supervision. The interview of each participant was done in a quiet location.

### Data quality control

The project team members developed the data collection tool in English, translated it into the local language, Amharic, and then retranslated it back into English by a language expert to ensure data quality. Data collectors and supervisors were trained for 3 days regarding the tool, consent and confidentiality, data collection techniques and approaches, and other important issues before the actual data collection. A pretest was conducted on 5% of the total sample size outside the data collection site. Some amendments to the data collection tool, such as the order of the questions, were made after the pretest. Supervisors and researchers closely monitored each data collection site when collecting the data. The collected data were checked for their completeness during data collection by the investigators and supervisors. Once the data had been entered into data analysis software, the data were cleaned using the techniques of frequency analysis and cross-tabulation.

### Data processing and analyses

The data were entered into Epi Info version 7 software and transferred to the Social Science Statistical Package (SPSS) version 25 software. Before analysis, the data were cleaned using frequency, listing, and sorting to find any missing values. After that, corrections were made by changing the original questionnaire. The income status of the participants was analyzed using principal component analysis (PCA). A descriptive analysis was done and reported using numbers and percentages. The mean and standard deviation (SD) measures of the summary were used. The result was presented using texts and tables. Variables with a *p* < 0.2 in bivariable logistic regression were entered into the multivariable binary logistic regression model. Finally, a multivariable binary logistic regression model was used to assess the association between dependent and independent variables. A *P* < 0.05 and the adjusted odds ratio (AOR) with a 95% CI were used to declare statistically significant predictors in multivariable analysis. Prior to multivariable logistic regression analysis, the presence of multicollinearity between the independent variables was checked using the variance inflation factor (VIF), and there is no multicollinearity between independent variables. The Hosmer and Lemeshow test was used to diagnose the model's fitness, and the model was adequate.

## Result

### Sociodemographic characteristics

A total of 1,597 participants took part in the study. The mean age of the respondents was 19.18 (SD ± 2.83) years. Out of the participants, 817 (51.2%) were women, and 854 (53.5%) were from rural residences. Most (92.9%) of the participants were Orthodox Christian, and more than half (60.6%) were students. More than three-fourths (79.6%) of respondents were single, and 758 (47.5%) of them were living with their mother and father. In addition, 1,147 (71.8%) participants had no personal income (Table 1).

**Table 1:** Sociodemographic characteristics of youths living in the central, west, and north Gondar zones.

**Variable**	**Frequency (*n*)**	**Percent (%)**
**Age (years)**		
15–19	869	54.4
20–24	728	45.6
**Sex**		
Male	780	48.8
Female	817	51.2
**Residence**		
Rural	854	53.5
Urban	743	46.5
**Religion**		
Orthodox Christian	1,483	92.9
Muslim	104	6.5
Other	10	.6
**Marital status**		
Single	1,272	79.6
Married	283	17.7
Divorced	42	2.6
**Educational status**		
Have no formal education	182	11.4
Primary school (1–8)	671	42.0
High school (9–10)	385	24.1
Preparatory school (11–12)	165	10.3
College and university	194	12.1
**Occupation**		
Job seeker	225	14.1
Student	968	60.6
Merchant	404	25.3
**Living with**		
Alone	213	13.3
Both father and mother	758	47.5
Mother or father only	229	14.3
Husband/wife	222	13.9
Sister/brother	105	6.6
**Mother alive**		
Yes	1,419	88.9
No	178	11.9
**Father alive**		
Yes	1,283	80.3
No	314	19.7
**Personal income/month**		
Yes	450	28.2
No	1,147	71.8
**Amount of personal income**		
50–1,800 Ethiopian Birr	350	79.6
>1,800 Ethiopian Birr	90	20.4
**Family wealth status**		
Poorest	319	19.9
Poor	320	20.1
Middle	319	19.9
Rich	320	20.1
Richest	319	19.9

### Clinical and social factors

Among the participants, 398 (24.9%) had common mental health problems. Approximately 91 (5.7%) of participants had a comorbid medical illness, of which kidney-related disease was the most common, 57 (60.4%). Regarding social factors, 449 (21.5%) of the participants reported an incident of violence in the previous 12 months (Table 2).

**Table 2:** Clinical and social characteristics of youths living in the central, west, and north Gondar zones.

**Variable**	**Frequency (*n*)**	**Percent (%)**
**Common mental health problem**		
No	1,199	75.1
Yes	398	24.9
**Chronic illness**		
No	1,506	94.3
Yes	91	5.7
**Types of chronic illness**		
HTN	22	22.2
DM	17	17.2
Renal-related disease	60	60.6
**Violence in the last 12 months**		
No	1,254	78.5
Yes	343	21.5
**Sexual violence in the last 12 months**		
No	1,543	96.6
Yes	54	3.4
**Physical violence in the last 12 months**		
No	1,471	92.1
Yes	126	7.9
**Emotional violence in the last 12 months**		
No	1,380	86.4
Yes	217	13.6
**Risky sexual practices**		
No	1,158	72.5
Yes	439	27.5

### Substance use characteristics

From the total number of individuals involved in the study, 3.6, 70.3, and 5.0% had used tobacco, alcohol, and khat, respectively, at least once in their lifetime. Moreover, 46 (2.9%) of participants used other abusive substances, and among all users, 38 (2.4%) used tobacco currently (Table 3).

**Table 3:** Substance use characteristics of youths living in the central, west, and north Gondar zones.

**Variable**	**Frequency (*n*)**	**Percent (%)**
**Previous substance use**		
No	475	29.7
Yes	1,122	70.3
**Previous alcohol use**		
No	475	29.7
Yes	1,122	70.3
**Previously chewing khat**		
No	1,517	95.0
Yes	80	5.0
**Previously smoking a cigarette**		
No	1,540	96.4
Yes	57	3.6
**Currently smoking cigarettes**		
No	1,559	97.6
Yes	38	2.4

### Magnitude of a suicide attempt

The lifetime prevalence of suicide attempts in the study participants was 5.5 (95% CI: 4.4, 6.6%), and 44 (2.8%) of them had reported a suicide attempt in the 3 months before the data collection time. The prevalence of lifetime suicidal ideation was 9.9% (95% CI: 8.6, 11.4%). Among those who had a history of suicidal ideation, 75 (47.5%) had thought about ending their life in the previous month (Table 4).

**Table 4:** Magnitude of suicide attempts among youths living in the central, west, and north Gondar zones.

**Variable**	**Frequency (*n*)**	**Percent (%)**
**Lifetime suicide attempt**		
No	1,509	94.5
Yes	88	5.5
**Suicide attempts in the last 3 months**		
No	44	50
Yes	44	50
**Lifetime suicidal ideation**		
No	1,439	90.1
Yes	158	9.9
**Suicidal ideation in the last 1 month**		
No	83	52.5
Yes	75	47.5

### Determinants of a suicide attempt

In a multivariable analysis, the odds of attempting suicide among participants with risky sexual practices were almost two times higher compared to participants without risky sexual practices (AOR = 1.89, 95% CI: 1.19–2.99). The presence of common mental health problems was significantly associated with a suicide attempt in the study. Participants with common mental health problems were six times more likely to attempt suicide compared to those who did not have a mental health problem (AOR = 6.31, 95% CI: 3.78–10.88). The rate of having attempted suicide among participants who had personal income was 1.7 times higher than among participants who had no personal income (AOR = 1.68, 95% CI: 1.05–2.71). Youths who had experienced violence in the previous 12 months were almost three times more likely to have attempted to end their lives (AOR = 2.81, 95% CI 1.76–4.49**)** than those who had not experienced violence in the previous 12 months (Table 5).

**Table 5:** Logistic regression analysis of factors associated with suicide attempts among youths living in the central, west, and north Gondar zones.

**Variable**	**Suicide attempt**			**Odds ratio (95% CI)**		
	**Yes**	**No**	**Crude (COR)**	**Adjusted (AOR)**
**Age**				
15–19	42	827	0.75 (0.49–1.16)	1.13 (0.69–1.84)
20–24	46	682	1	1
**Occupation**				
Looking for work	14	211	0.72 (0.37–1.37)	0.89 (0.42–1.89)
Student	40	928	0.46 (0.29–0.75)	0.80 (0.42–1.52)
Merchant	34	370	1	1
**Marital status**				
Single	60	1,212	1	1
Married	19	264	1.45 (0.83–2.47)	0.79 (0.42–1.49)
Divorced	9	33	5.50 (2.52–12.03)	1.88 (0.77–4.59)
**Personal income**				
No	54	1,093	1	1
Yes	34	416	1.65 (1.06–2.57)	1.68 (1.05–2.71)^*^
**Common mental health problem**				
No	24	1,175	1	1
Yes	64	334	9.94 (5.64–17.50)	6.31 (3.78–10.88)^***^
**Violence**				
No	49	1,214	1	1
Yes	72	295	4.94 (3.19–7.66)	2.81 (1.76–4.49)^***^
**Previous substance use**				
No	16	459	1	1
Yes	72	1,050	1.97 (1.13–3.42)	1.34 (0.75–2.39)
**Risky sexual practices**				
No	42	1,116	1	1
Yes	46	393	3.11 (2.02–4.79)	1.89 (1.19–2.99)^**^

* p-value is significant at p < 0.05,

** p-value is significant at p < 0.01.

*** p-value is significant at p < 0.001, p-value in the Hosmer and Lemeshow test = 0.807.

## Discussion

This study investigated the lifetime prevalence of suicide attempts among 1,597 youths in 2019 Gregorian calendar (GC). The prevalence of lifetime suicide attempts was 5.5% (95% CI: 4.4, 6.6%). The prevalence of lifetime suicide attempts in this study was in line with the studies conducted in Bahirdar 7.6% (23) and Gondar 7.4% (24). However, it was higher than the results from the studies done in Vietnam (3.8%) (25) and India (0.33%) (26). This might be due to differences in study settings and sociocultural characteristics as well as differences in the study population; for example, an Indian study included participants aged <15 years, an age group in which suicide attempts exhibit a relatively low prevalence.

In contrast, the result was lower than the findings from the studies done in Danigla, northwest Ethiopia, 16.2%, Addis Ababa, 13.9% (17), and Gondar, northwest Ethiopia, 19.2% (27). This discrepancy might be attributable to differences in the study population and participants' health statuses. For example, the studies in Addis Ababa and Gondar were carried out among patients with HIV and psychiatric disorders, and patients with psychiatric disorders are high-risk individuals compared to individuals in community-based studies (23). HIV positive patients may have a decreased quality of life, which may lead them to think of death (17), or high HIV infection-related stigmatization may lead them to end their lives due to the feelings of isolation, loneliness, and uncertainty about their future lives (24).

Regarding factors associated with suicide attempts, the result of this study showed that participants with common mental health problems were more likely to attempt suicide compared to those who did not have mental health problems. This finding is supported by studies done by Debre Markos and Gondar (21, 23). This may be because of depression and feelings of hopelessness, guilt, and worthlessness that may drive them to attempt suicide. This finding indicated the need for psychosocial support for young people and integration with youth-friendly health services to promote better mental health. Working on young people's mental health would help prevent suicide ideation and attempts.

Participants with a history of violence in the past 12 months were three times more likely to attempt suicide compared to those without this experience. This finding is in line with a study done in Danigla, northwest Ethiopia (6). This might be because sexual and physical abuse increased the risk of common mental disorders, and sexual violence increased the occurrence of post-traumatic stress disorder (28), or it might be to escape from physical injury. This implicates the need for psychosocial support following the experience of violence and to ensure the integration of psychological treatment into medical treatment programs for those who have experienced violence.

The rates of attempting suicide among participants with risky sexual practices was almost two times higher compared to participants without risky sexual practices. This might be because risky sexual practices increase the risk of an unplanned pregnancy, which causes fear and stress that may force the individual to end their life. This finding indicated the need for sex education programs that combine pregnancy information for youths and promote sexual and reproductive health in youths. Participants who had personal income were more likely to attempt suicide compared to those who had no personal income. This might be because youths who have personal income tend to develop substance abuse because these youths have the money to buy substances, or that they have a job which brings them stress and therefore they turn to substances; additionally, those using psychoactive substances (like khat) are vulnerable to thinking about suicide due to mental health problem.

## Limitations of this study

Self-reported lifestyle behaviors and underlying mental and chronic medical conditions may have been under-reported. Individuals may not have disclosed their suicide attempts through an interview, and the lifetime frequency of suicidal ideation and suicide attempts could be under-reported due to recall bias.

## Conclusion

The prevalence of suicide attempts among youth was found to be low. The presence of common mental health problems, risky sexual practices, having a personal income, and a history of violence increase the likelihood of suicide attempts. Working on the reduction of risky sexual practices, ensuring the integration of psychological treatment into medical treatment programs for victims of violence, providing psychosocial support for young people, and integrating youth-friendly health services to promote mental health would help to reduce suicide attempts among youth.

## Data availability statement

The raw data supporting the conclusions of this article will be made available by the authors, without undue reservation.

## Ethics statement

The studies involving human participants were reviewed and approved by the Ethical review board of the University of Gondar (reference number O/V/P/RCS/05/428/2018). A support letter was obtained from the Research, Technology Transfer, and Community Service Directorate, College of Medicine and Health Science. Permission letters were secured from each zonal health department and district health office. For participants under the age of 18, written informed consent from the parents or guardian and assent from the participant was obtained. Informed consent was also secured from each participant age 18 years and above. Their name and other forms of personal identification were not entered into the data collection format to maintain confidentiality. When we found serious suicide attempt history or plan (if the authors met cases) we communicate with their family and linked to the clinic then they get psychological support.

## Author contributions

ABG, AAG, HY, TA, YH, SA, and EB engaged in proposal and tool development and compilation, provided training for data collectors, and supervised the data collection process. EF was involved in data management and analysis and wrote the manuscript. All authors revised, read, and approved the manuscript.
